# Abdominal obesity, gender and the risk of rheumatoid arthritis – a nested case–control study

**DOI:** 10.1186/s13075-016-1171-2

**Published:** 2016-11-29

**Authors:** Lotta Ljung, Solbritt Rantapää-Dahlqvist

**Affiliations:** Department of Public Health and Clinical Medicine/Rheumatology, Umeå University, University Hospital, Umeå, SE-901 87 Sweden

**Keywords:** Rheumatoid arthritis, Epidemiology, Risk factors, Lifestyle, Obesity, Case–control

## Abstract

**Background:**

The risk of development of rheumatoid arthritis (RA) could be affected by immune activation in obesity. Our objective was to evaluate the association between obesity in general, and abdominal obesity, and the risk for subsequent development of RA.

**Methods:**

In two large population-based, prospective cohorts, 557 cases (mean age at RA symptom onset 58, SD 10 years, 68% women) who subsequently developed RA and 1671 matched controls were identified. From a health examination antedating symptom onset (median 5.5 years), collected data on body mass index (BMI; kg/m^2^), smoking habits, and educational level was used in conditional logistical regression models. Corresponding regression models were used to analyse the association between waist circumference measurements (cm) and RA development in a subset of the population.

**Results:**

BMI and waist circumference were associated with the risk of RA development, adjusted odds ratio (OR) (95% CI), 1.13 (1.00, 1.28) per 5 kg/m^2^, and 1.02 (1.01, 1.04) per cm, respectively. An association was also observed for obesity (BMI ≥30) OR 1.45 (1.07, 1.95), compared with BMI <25. After stratification for sex the associations were enhanced in men, and attenuated in women. Among men with BMI above normal a 3–5 times increased risk for RA disease development at 50 years of age or earlier was observed. Abdominal obesity with waist circumference >102 cm was associated with a 2–3 times increased risk of RA, but not abdominal obesity (>88 cm) in women.

**Conclusions:**

Obesity or abdominal obesity, respectively, was independently associated with a modest increase of the risk for subsequent development of RA. This appeared to be relevant mainly for early RA disease onset among men.

**Electronic supplementary material:**

The online version of this article (doi:10.1186/s13075-016-1171-2) contains supplementary material, which is available to authorized users.

## Background

At the time point when the first symptoms indicating the onset of rheumatoid arthritis (RA) become apparent for the individual, the immune system has been activated for several years. This has been demonstrated by the presence of anti-citrullinated peptide antibodies (ACPA), rheumatoid factor (RF) and up-regulation of cytokines and chemokines in blood samples [1, 2]. The pathogenesis of RA is largely unknown but heritability of approximately 50% for ACPA-positive and approximately 20% for ACPA-negative RA has been shown, and environmental factors are believed to interplay with genetic factors during the early, pre-symptomatic stages [3]. Smoking and periodontal disease have been linked to development of mainly seropositive RA [4, 5]. Obesity could be another possible environmental risk factor due to the pro-inflammatory nature of the adipose tissue, but studies evaluating the association between body mass index (BMI) and the risk of RA have shown contradictory results [6–16].

Several studies have reported a positive association between obesity and the risk of RA development, overall [8, 9, 11, 12, 16], in women [6, 10, 16], and in seronegative RA disease development [9, 15]. Negative associations with obesity have been reported in two studies: in RA [7] and ACPA-positive RA [15], respectively, in men. Another two studies reported no statistically significant associations between BMI or BMI categories and RA [13, 14]. The comparison between studies is hampered by differences in methodology, but a recent meta-analysis suggested a 30% increase in the risk of RA overall in obesity compared with normal weight [17].

Generally, BMI based on weight and height measurements has limitations, as the proportion of body fat and the location of the adipose tissue are of importance for the risks associated with obesity. Visceral adipose tissue depots are associated with metabolic disease, whereas subcutaneous adipose tissue might have a protective effect on cardiovascular risk factors [18, 19]. Men with RA have been shown to have increased deposition of intra-abdominal fat and the proportion of visceral fat is associated with cardiovascular risk factors [20]. Moreover, differences have been observed between men and women in body fat distribution or the associations between adipose tissue and comorbidity [18–21].

More profound knowledge of the tentative role of obesity in the phases of RA before symptom onset could improve the possibility of RA disease prevention in high-risk individuals. Our aim was to investigate the association between obesity as measured by BMI or waist circumference and subsequent development of RA, taking gender into account, in a case–control study nested within a large population-based, prospective cohort.

## Methods

Two previously described cohorts, the Västerbotten Intervention Programme (VIP) and the Northern Sweden Multinational Monitoring of Trends and Determinants in Cardiovascular Disease (MONICA) project, were used for this nested case–control study [22, 23]. In brief, the VIP was started in 1985, and since 1991 all inhabitants of Västerbotten County in northern Sweden have been invited to participate in VIP by having health examination visits at 40, 50 and 60 years of age, mainly for screening of cardiovascular risk factors and prevention of cardiovascular morbidity. The Northern Sweden MONICA project has performed population risk factor surveys six times since 1986 in individuals aged 25–74 years. Both cohorts are characterized by a high participation rate (70–80%) and data on life style factors are collected in a similar manner in both, making merging of data from the two cohorts possible. Demographic and anthropometric data and data on cardiovascular risk factors are collected through standardised measurements of weight (kg), height (cm) and blood pressure, and through blood sampling and questionnaires. Waist circumference (cm) is measured in the MONICA cohort, and was introduced as a measurement in VIP in 2004.

In Västerbotten County, with no private practitioners in rheumatology, the only rheumatology clinic is the University hospital, to which all individuals with newly diagnosed or suspected RA are referred. In this setting we identified all known patients with RA according to the American Rheumatism Association (ARA) 1987 classification criteria [24], and who had one or more registered visit 1986–2013 as a participant in the VIP and/or MONICA cohorts before the date of onset of RA symptoms (index date). Data from the health examinations were retrieved (VIP, n = 508, and MONICA, n = 49), and for every case, three controls (n = 1671) matched for age, sex, cohort, year of health examination, and area of inhabitance (rural/urban), were randomly selected from the cohorts. For cases with more than one health examination (n = 141) the latest visit antedating RA symptom onset was used.

Rheumatoid factor (RF) and ACPA at/after diagnosis were analysed using clinical routine methods and the anti-cyclic citrullinated peptide 2 (anti-CCP2) test (Eurodiagnostica, Malmö, Sweden), respectively. The exposures were assessed as BMI (kg/m^2^) and waist circumference (measured in centimetres, available for 138/557 cases), respectively, as registered at the health examinations. Abdominal obesity was identified as waist circumference >102 cm in men and >88 cm in women [25]. Body mass index (BMI) was categorised according to the World Health Organisation (WHO) criteria: obesity (≥30 kg/m^2^), overweight (25–29.99 kg/m^2^), normal weight (18.5–24.99 kg/m^2^), or underweight (<18.5 kg/m^2^). Information on smoking habits and level of education was retrieved from the questionnaire data.

### Statistical methods

Descriptive data for cases and controls were summarized and presented as proportions and means as appropriate. Odds ratios for the risk of subsequent development of RA associated with BMI, waist circumference, BMI categories or the presence of abdominal obesity, were calculated in conditional logistic regression models with additional adjustment for smoking habits and educational level. With <1% underweight cases and controls, the two lowest BMI categories were combined and BMI <25 kg/m^2^ was used as the reference in analyses evaluating BMI categories. Analyses stratified for sex, age at RA symptom onset, and subsequent seropositive/seronegative RA disease were performed. In a sensitivity analysis in women the OR for RA associated with abdominal obesity using the same definition as in men (>102 cm) was analysed. Another sensitivity analysis was performed to evaluate the risk associated with obesity (BMI ≥30 kg/m^2^) in the presence/absence of abdominal obesity (identified by waist circumference) among both cases and controls. In this analysis binary logistic regression models were used, with adjustments for age, sex, year of health examination, cohort, smoking and level of education, as the matching could not be maintained in this analysis. In the stratified analyses the number of individuals in each stratum are presented in Additional file 1: Tables S1–S3. Multiplicative interaction was assessed by adding an interaction term to the regression models, and additive interaction by the method outlined by Zou [26]. Statistical calculations were performed using IBM SPSS Statistics version 23 (IBM SPSS, IBM Corp, NY, USA).

## Results

The mean age (standard deviation, SD) at the time point for the health examination was 51.9 (9.1) years and 68% were women (Table 1). The mean (SD) time period before symptom onset was 6.5 (4.5) years and the mean age at symptom onset was 58.3 (10.5) years (Table 1). Smoking and a low level of education were more common among cases, *p* < 0.001 and *p* < 0.01, respectively (Table 1). Most cases (434/552, 85.6%) developed seropositive disease (Table 1); equally so for men and women (84.2% and 86.2%, respectively). The proportion of seropositive disease was comparable in groups based on quartiles of age at onset of RA symptoms (*p* = 0.152); >90% of the cases (men and women) in the percentile with RA symptom onset <50 years of age developed seropositive RA disease.

**Table 1:** Characteristics of included cases and controls

	Cases (n = 557)	Controls (n = 1671)
Female sex, *n* (%)	379 (68)	1137 (68)
Data from the health examination visit		
Age at examination (years), mean (SD)	51.9 (9.1)	51.9 (9.1)
Body mass index (kg/m^2^), mean (SD)	26.3 (4.3)	25.9 (4.0)
BMI category, *n* (%)		
Underweight	5 (0.9)	15 (0.9)
Normal weight	220 (39.7)	748 (45.3)
Overweight	231 (41.7)	661 (40.1)
Obese	98 (17.7)	226 (13.7)
Smoking habits, *n* (%)		
Non-smoker	177 (32.2)	812 (49.6)
Ex-smoker	175 (31.9)	498 (30.4)
Smoker	197 (35.9)	327 (20.0)
Educational level, *n* (%)		
9 years or less	219 (39.8)	538 (32.8)
10–12 years	239 (43.5)	757 (46.1)
More than 12 years	92 (16.7)	346 (21.1)
Waist circumference (cm), mean (SD) (sample size^a^: cases n = 138, controls n = 409)	92 (14)	89 (12)
Abdominal obesity^b^, *n* (%) (sample size^a^: cases n = 138, controls n = 409)	54 (39.1)	131 (32.0)
Positive ACPA at examination, *n* (%) (sample size^c^: cases n = 476, controls n = 800)	179 (37.6)	20 (2.5)
Data from the time point of RA symptom onset or later (only cases)		
Time between the health examination visit and the onset of RA symptom (years)		
Mean (SD)	6.4 (4.5)	
Median (Q_1_, Q_3_)	5.5 (2.9, 8.9)	
Age at RA symptom onset (years), mean (SD)	58.3 (10.5)	
Positive ACPA at/after RA diagnosis, *n* (%) (sample size^c^: cases n = 473)	380 (80.4)	
Positive RF at/after RA diagnosis, *n* (%) (sample size^c^: cases n = 552)	434 (78.6)	
Positive ACPA and/or RF at/after RA diagnosis, *n* (%) (sample size^c^: cases n = 554)	474 (85.6)	

^a^Waist circumference was measured in the MONICA cohort, and was introduced as a measurement in the VIP cohort 2004. ^b^Abdominal obesity was defined as waist circumference >102 cm (men) or >88 cm (women). ^c^ACPA analysed in blood samples from the health examination was available for 476 cases and 800 controls. Anti-citrullinated peptide antibodies (ACPA) or rheumatoid factor (RF) at/after rheumatoid arthritis (RA) diagnosis was available for 473 cases and 552 cases, respectively, and either ACPA or RF at/after diagnosis was available for 554 cases. *SD* standard deviation, *BMI* body mass index

In a conditional regression model comprising all cases and controls BMI was associated with the risk of development of RA: crude odds ratio (OR) 1.14 (95% confidence interval, 1.01, 1.28) for every 5 kg/m^2^ increase in BMI, and after adjustment for smoking habits and level of education, OR 1.13 (1.00, 1.28) (Table 2). Analyses stratified by sex resulted in similar, but not statistically significant associations (Table 2).

**Table 2:** Associations between overweight or obesity and risk of development of rheumatoid arthritis

		All	Men	Women
		OR (95% CI)	OR (95% CI)	OR (95% CI)
All	BMI (OR per 5 kg/m^2^ increase in BMI)	1.13 (1.00, 1.28)	1.17 (0.90, 1.50)	1.13 (0.98, 1.30)
	BMI category vs. low-normal weight			
All	Obesity	1.45 (1.07, 1.95)	1.78 (1.01, 3.12)	1.37 (0.96, 1.95)
	Overweight	1.21 (0.96, 1.52)	1.29 (0.84, 1.96)	1.21 (0.92, 1.59)
	Normal-underweight	Reference	Reference	Reference
Population divided by the age of the case at RA symptom onset:				
≤50 years	Obesity	1.89 (1.02, 3.52)	4.63 (1.14, 18.8)	1.39 (0.68, 2.87)
	Overweight	1.43 (0.89, 2.29)	2.90 (1.08, 7.76)	1.20 (0.68, 2.10)
	Normal-underweight	Reference	Reference	Reference
51–59 years	Obesity	1.37 (0.76, 2.47)	1.93 (0.68, 5.50)	1.16 (0.56, 2.40)
	Overweight	1.10 (0.71, 1.72)	1.02 (0.47, 2.25)	1.23 (0.72, 2.13)
	Normal-underweight	Reference	Reference	Reference
60 − 65 years	Obesity	1.62 (0.91, 2.89)	1.06 (0.35, 3.23)	1.96 (0.98, 3.92)
	Overweight	1.08 (0.68, 1.71)	0.60 (0.25, 1.46)	1.28 (0.73, 2.25)
	Normal-underweight	Reference	Reference	Reference
≥66 years	Obesity	1.14 (0.61, 2.14)	1.15 (0.32, 4.16)	1.20 (0.58, 2.48)
	Overweight	1.29 (0.81, 2.01)	1.68 (0.67, 4.21)	1.17 (0.67, 2.05)
	Normal-underweight	Reference	Reference	Reference
Population divided by the serologic status for the case after RA diagnosis:				
Seropositive RA	Obesity	1.40 (1.00, 1.96)	1.33 (0.71, 2.50)	1.47 (0.99, 2.18)
	Overweight	1.27 (0.99, 1.63)	1.15 (0.73, 1.84)	1.34 (0.99, 1.81)
	Normal-underweight	Reference	Reference	Reference
Seronegative RA	Obesity	1.76 (0.88, 3.52)	12.3 (2.14, 71.1)	1.07 (0.47, 2.46)
	Overweight	1.93 (0.51, 2.15)	3.44 (0.82, 14.5)	0.59 (0.27, 1.30)
	Normal-underweight	Reference	Reference	Reference

Analyses of the risk of development of rheumatoid arthritis (RA) associated with body mass index (BMI) for every 5 kg/m^2^ increase, or BMI categories of overweight or obesity compared with normal-underweight. Results are presented in subjects overall, and divided by sex. Results from conditional logistic regression, adjusted for education and smoking, in case–control sets matched for age, sex and year of examination. Analyses of BMI categories were also performed after dividing the case–control sets by quartiles of the age of the case at onset of RA symptoms, or whether the case subsequently developed seropositive or seronegative RA disease. *OR* odds ratio

### Analyses of risk associated with BMI categories overweight or obese

In models evaluating BMI, the category for obesity (OR 1.48 (1.12, 1.97)), but not for being overweight (OR 1.20 (0.96, 1.49)), was found to be associated with development of RA. Adjustments for smoking and level of education had a low impact on the estimates: the adjusted OR for obesity was 1.45 (1.07, 1.95) and for being overweight it was 1.21 (0.96, 1.52) (Table 2). No interactions was found between BMI and smoking (data not shown).

In stratified analyses, adjusted for smoking and level of education, statistically significant associations were observed between obesity and RA symptom onset ≤50 years of age (OR 1.89 (1.02, 3.52)), and with seropositive disease (OR 1.40 (1.00, 1.96)) (Table 2).

Among men an association between obesity and RA development was observed overall in men in the lowest quartile of age at RA symptom onset, and for seronegative RA, respectively (Table 2). BMI above normal in men was associated with 3–5 times higher risk of developing RA at an early age (Table 2). The OR for development of seronegative RA among men was high, although based on a small number of individuals (see Additional file 1: Table S1). No statistically significant association was observed among women (Table 2).

### Analyses of the subset with waist circumference measurements

Waist circumference measurement was available in a subset comprising 138 cases and 409 controls; 68% were women. As the measuring of waist circumference was introduced in the health examinations in VIP in later years, individuals with waist circumference measurements had been included at a later time point than individuals without this measurement, with median year of examination 2004 and 1996, respectively (see Additional file 1: Table S4). Among the cases 54 (39.1%) had abdominal obesity, compared with 131 (32.0%) of the controls. Abdominal obesity was more common among women; 38 (40.0%) of cases and 104 (37.3%) of the female controls had waist circumference >88 cm.

Analyses of the risk of RA associated with waist measurement indicated a 2% increase in the risk of RA development for each centimetre increase in waist measurement (adjusted OR 1.02 (1.01, 1.04)) overall. The risk was enhanced in men (adjusted OR 1.05 (1.01, 1.09)). No statistically significant association was observed in women (Table 3). Abdominal obesity (using the cutoff >88 and >102 cm in women and men, respectively) overall was not statistically significantly associated with RA development (unadjusted OR 1.39 (0.93, 2.09), OR adjusted for smoking and level of education 1.40 (0.92, 2.14)) (Table 3).

**Table 3:** Associations between obesity and the risk of development of rheumatoid arthritis in the subset with available waist circumference measurement

	All	Men	Women	Development of seropositive RA	Development of seronegative RA
	OR (95% CI)	OR (95% CI)	OR (95% CI)	OR (95% CI)	OR (95% CI)
BMI (OR per 5 kg/m^2^)	1.31 (1.05, 1.64)	1.91 (1.15, 3.19)	1.19 (0.92, 1.53)	1.28 (0.99, 1.65)	1.52 (0.88, 2.64)
Waist circumference (OR per cm)	1.02 (1.01, 1.04)	1.05 (1.01, 1.09)	1.01 (0.99, 1.04)	1.02 (1.00, 1.04)	1.02 (0.98, 1.07)
Abdominal obesity	1.40 (0.92, 2.14)	3.57 (1.50, 8.51)	1.04 (0.63, 1.71)	1.36 (0.85, 2.17)	1.21 (0.41, 3.62)
No abdominal obesity	Reference	Reference	Reference	Reference	Reference
Obesity	2.95 (1.20, 3.51)	3.56 (1.27, 9.96)	1.68 (0.88, 3.23)	1.98 (1.07, 3.67)	2.18 (0.62, 7.67)
Overweight	0.84 (0.52, 1.36)	1.12 (0.41, 3.04)	0.77 (0.44, 1.35)	1.02 (0.60, 1.74)	0.31 (0.09, 1.07)
Normal or underweight	Reference	Reference	Reference	Reference	Reference
Obesity	2.12 (1.35, 3.63)	3.37 (1.37, 8.31)	1.88 (1.02, 3.46)	1.96 (1.12, 3.45)	3.66 (1.15, 11.7)
No obesity	Reference	Reference	Reference	Reference	Reference

Associations between abdominal obesity (measured by waist circumference) or being overweight and/or obese (as assessed by body mass index (BMI)) and the risk of onset of rheumatoid arthritis (RA) in all individuals, men and women separately, or divided based on whether the patients (cases) later developed seropositive or seronegative RA. In the subset the individuals with available measurements of waist circumference (from the MONICA cohort and from the VIP cohort from 2004 and onwards) were included. Results from conditional logistic regression analyses are presented adjusted for education and smoking, in case–control sets matched for age, sex, cohort, and year of health examination. Abdominal obesity was defined as a waist circumference ≥102 cm (men) or ≥88 cm (women). Obesity was defined as BMI ≥30 kg/m^2^, and overweight as BMI >25 and <30 kg/m^2^. *OR* odds ratio

A three-fold increase in the risk of RA development was observed in men with abdominal obesity (Table 3). No association was observed among women (Table 3). In this subset BMI was associated with RA overall, and particularly in men (Table 3). Obesity defined as BMI ≥30 kg/m^2^ compared with BMI <25 kg/m^2^ was associated with the risk of RA development, overall, in men and in seropositive RA disease, with risk estimates indicating double to triple increased risk (Table 3). Positive associations with RA development overall were observed in both sexes and in both seropositive and seronegative disease when comparing obesity with non-obesity (Table 3).

### Sensitivity analyses

Using the same cutoff value for abdominal obesity in women as in men (waist circumference >102 cm), 18 (18.9%) of the female cases and 26 (9.3%) of the controls were abdominally obese. Use of this definition resulted in a model where abdominal obesity in women was associated with double the risk of RA development (Table 4).

**Table 4:** Risk for rheumatoid arthritis associated with abdominal obesity, defined as waist circumference >102 cm, in women

	Women
	OR (95% CI)
All	2.28 (1.15, 4.55)
Divided by the serostatus of the case after RA diagnosis:	
Seropositive RA	2.54 (1.18, 5.47)
Seronegative RA	1.44 (0.29, 7.16)

Sensitivity analyses of the associations between abdominal obesity in women (n = 95 cases, and 279 controls), using the same cutoff as in men (waist circumference >102 cm), and risk of onset of rheumatoid arthritis (RA). Analysed overall and divided by serological status for rheumatoid factor and/or anti-citrullinated peptide antibodies for the case after RA diagnosis, respectively. Results from conditional logistic regression analyses adjusted for education and smoking, in case–control sets matched for age, year of examination and cohort

In an analysis evaluating abdominal obesity and obesity identified by BMI in the same model, obesity (BMI ≥30 kg/m^2^) was associated with RA among individuals with abdominal obesity, but not among those with a normal waist circumference (Table 5).

**Table 5:** Association between obesity and risk of rheumatoid arthritis evaluated in presence/absence of abdominal obesity

		All
		OR (95% CI)
With abdominal obesity	Obesity (BMI ≥30 kg/m^2^)	2.66 (1.26, 5.64)
	Not obesity (BMI <30 kg/m^2^)	Reference
No abdominal obesity	Obesity (BMI ≥30 kg/m^2^)	0.48 (0.05, 4.40)
	Not obesity (BMI <30 kg/m^2^)	Reference

Sensitivity analysis of the association between obesity and the risk of onset of rheumatoid arthritis in individuals divided on presence/absence of abdominal obesity. Results from logistic regression models are presented adjusted for age, sex, year of examination, cohort, education and smoking habits. Abdominal obesity was defined as a waist circumference ≥102 cm (men) or ≥88 cm (women). Obesity was defined as body mass index (BMI) ≥30 kg/m^2^

## Discussion

Several previous studies have shown positive associations between obesity and RA development, mostly of moderate strength and in various strata of age, sex, and serological status [6, 8–12, 15, 16]. A recent meta-analysis identified a pooled risk estimate of 1.31, which is in parity with our fully adjusted estimate of 1.45 [17].

Age at disease onset has rarely been considered before, but the results from the Nurses’ Health Study indicated a stronger association between obesity and RA development at a younger age [6]. In the present study the association between obesity and RA was seen mainly for early disease onset among men, and the Nurses’ Health Study included women exclusively, which makes the results difficult to compare [6]. Interestingly, our results contradict the results from a Swedish study in which high BMI in some regression models were associated with a reduced risk of RA in men, who compared with our study, were examined at a higher age [7]. If obesity acts as a strong trigger for disease onset at an early age in predisposed men, the selection of older age at examination could theoretically have contributed to an apparent protective effect. An apparently protective effect of obesity on development of seropositive RA among men was presented in the Swedish EIRA study [15]. The differences between the present study and the other two studies might be attributed to methodological differences, such as study design, data collection, or participation rates, or specific characteristics of the populations.

Our findings were further emphasised by the association between waist circumference and RA development overall and in men, to our knowledge not previously evaluated or reported. A greater proportion of intra-abdominal body fat, in which the production and secretion of proinflammatory substances is most abundant, has been suggested as a reason for more negative consequences of obesity in men compared with women [21]. Extreme abdominal obesity in women, as evaluated in the sensitivity analyses, was associated with RA development, indicating that the distribution of fat and/or proportion of visceral fat might be of importance also in women. The subset of the study population with waist circumference differed from the group who did not have this measurement in several respects that often can be linked to the difference in calendar time for the health examination. In the more contemporary subset the impact of obesity seemed to be stronger, and statistically significant positive associations were observed in both sexes and in both seropositive and seronegative disease, depending on exposure definition.

The results indicate that abdominal obesity confers extra information to the presence of obesity in determining the risk of RA. It also indicates that cutoff values for estimation of cardiovascular risk might not be ideal for RA risk calculations. Differences in fat distribution (comparing individuals with similar BMI) or between life style factors in different populations (i.e. geographical regions), between the sexes, during aging, and over calendar time, could hypothetically contribute to the conflicting results from previous studies. As previously mentioned methodological differences make it difficult to perform direct comparisons between studies in this field.

In the present study we could not find support for a differentiated role for obesity according to the serologic status of RA, as some previous studies have indicated [7, 8, 15]. From this perspective we suggest that obesity, abdominal obesity and/or factors related to these, act as a trigger or drive on the inflammatory system(s), rather than exert a specific immunologic effect. Based on serological status, we found no reason to conclude that the RA disease that developed among men before 50 years of age constituted a specific subset. Presence of ACPA and/or RF was as common among the individuals who developed RA before 50 years of age as among cases with later onset. This was observed also among men, the stratum in which we found the strongest association between obesity and RA.

In spite of the strengths of the study, including the prospective, population-based cohort with standardised measurements that formed the basis of our study and the well-defined RA diagnoses, there are some limitations. The number of cases was low in some subgroups; it may therefore be questionable to draw any firm conclusions from the association between obesity and seronegative RA in men. It can also not be ruled out that unmeasured confounding or residual confounding could affect the results. Measurement of waist circumference was not available for all study participants. The health examinations were performed mainly at 10-year intervals from 40 years of age onwards, which limits the possibility of evaluating the exposure in individuals with disease onset at a young age, which is observed more often among women [27]. It also limits the possibility of longitudinal evaluation of BMI development before the onset of symptoms of RA.

## Conclusions

In conclusion, in this case–control study, nested within population-based prospective cohorts, we found obesity, and abdominal obesity, respectively, to be associated with a moderate increased risk of development of RA. This appeared to be most marked among men, and both obesity and being overweight were associated with early RA development in men. Clinically, interventions for obesity might be of importance for disease prevention in individuals with a high risk of disease development. In future research into environmental risk factors for RA, efforts should be made to include all ages and both sexes.

## Additional file

Additional file 1: Table S1. The number of individuals in each BMI category in total and as presented in the stratified analyses. **Table S2.** The number of individuals with or without abdominal obesity in total and as presented in the stratified analyses. **Table S3.** The number of female individuals with or without abdominal obesity defined as waist circumference >102 cm/ ≤102 cm. **Table S4.** Comparison of characteristics between the individuals in the subset with waist circumference measurement compared with the individuals without this measurement. (DOCX 25 kb)

## Abbreviations

ACPA: anti-citrullinated peptide antibodies
BMI: body mass index
MONICA: the Northern Sweden Multinational Monitoring of Trends and Determinants in Cardiovascular Disease
OR: odds ratio
RA: rheumatoid arthritis
RF: rheumatoid factor
SD: standard deviation
VIP: Västerbotten Intervention Programme
WHO: World Health Organisation
