# Supplementary material for: Abdominal obesity, gender and the risk of rheumatoid arthritis – a nested case–control study
Source: Arthritis Res Ther. 2016 Nov 29;18:277. doi: 10.1186/s13075-016-1171-2 (PMC5127000; doi:10.1186/s13075-016-1171-2)
Supplement: Additional file 1: Table S1. — The number of individuals in each BMI category in total and as presented in the stratified analyses. Table S2. The number of individuals with or without abdominal obesity in total and as presented in the stratified analyses. Table S3. The number of female individuals with or without abdominal obesity defined as waist circumference >102 cm/ ≤102 cm. Table S4. Comparison of characteristics between the individuals in the subset with waist circumference measurement compared with the individuals without this measurement. (DOCX 25 kb) [file 13075_2016_1171_MOESM1_ESM.docx]

## Additional file 1: Table S1

The number of individuals in each BMI category in total and as presented in the stratified analyses

|  | **All** | | **Men** | | **Women** | |  |
| --- | --- | --- | --- | --- | --- | --- | --- |
|  | **BMI category** | **Cases** | **Controls** | **Cases** | **Controls** | **Cases** | **Controls** |
| **All** | Obese  Overweight  Low or normal weight | 98  231  225 | 226  661  763 | 31  89  58 | 67  264  198 | 67  142  167 | 159  397  565 |
| **Age at RA symptom onset ≤50 years** | Obese  Overweight  Low or normal weight | 22  44  70 | 39  121  245 | 8  17  10 | 10  44  49 | 14  27  60 | 29  77  196 |
| **Age at RA symptom onset 51-59 years** | Obese  Overweight  Low or normal weight | 25  59  62 | 57  167  210 | 10  22  19 | 18  72  61 | 15  37  43 | 39  95  149 |
| **Age at RA symptom onset 60-65 years** | Obese  Overweight  Low or normal weight | 28  59  44 | 58  184  149 | 8  22  16 | 20  77  40 | 20  37  28 | 38  107  109 |
| **Age at RA symptom onset ≥66 years** | Obese  Overweight  Low or normal weight | 23  69  49 | 72  189  159 | 5  28  13 | 19  71  48 | 18  41  36 | 53  118  111 |
| **Seropositive RA** | Obese  Overweight  Low or normal weight | 77  200  194 | 188  559  658 | 23  72  54 | 60  216  167 | 54  128  140 | 128  343  491 |
| **Seronegative RA** | Obese  Overweight  Low or normal weight | 20  30  30 | 37  101  98 | 7  17  4 | 6  48  29 | 13  13  26 | 31  53  69 |

BMI – body mass index, RA – rheumatoid arthritis

## Additional file 1: Table S2

The number of individuals with or without abdominal obesity in total and as presented in the stratified analyses

|  | **All** | | **Men** | | **Women** | | **Development of seropositive RA** | | **Development of seronegative RA** | |
| --- | --- | --- | --- | --- | --- | --- | --- | --- | --- | --- |
|  | Cases | Controls | Cases | Controls | Cases | Controls | Cases | Controls | Cases | Controls |
| **Abdominal obesity**  **No abd. obesity** | 54  84 | 131  278 | 16  27 | 27  103 | 38  57 | 104  175 | 44  68 | 109  222 | 9  15 | 22  50 |
| **Obesity**  **Overweight**  **Normal -under** | 37  47  54 | 62  167  180 | 14  19  10 | 23  62  45 | 23  28  44 | 39  105  135 | 27  41  44 | 50  130  151 | 9  5  10 | 11  37  24 |
| **Obesity**  **No obesity** | 37  101 | 62  437 | 14  29 | 23  107 | 23  72 | 39  240 | 27  85 | 50  281 | 9  15 | 11  61 |

RA – rheumatoid arthritis

## Additional file 1: Table S3

The number of female individuals with or without abdominal obesity defined as waist circumference >102 cm/ ≤102 cm.

|  |  | **Women II** | |
| --- | --- | --- | --- |
|  | **Abdominal obesity** | **Cases** | **Controls** |
| **All** | No | 77 | 253 |
|  | Yes | 18 | 26 |
| **Seropositive RA** | No | 64 | 212 |
|  | Yes | 15 | 19 |
| **Seronegative RA** | No | 12 | 38 |
|  | Yes | 3 | 7 |

RA – rheumatoid arthritis

**Additional file 1: Table S4. Comparison of characteristics between the individuals in the subset with waist circumference measurement compared with the individuals without this measurement**

|  | **Cases with data on waist (n=138)** | **Cases without data on waist (n=419)** | **Controls with data on waist (n=409)** | **Controls without data on waist (n=1262)** | *Cases vs. cases, p* | *Controls vs. controls, p* | *Cases vs. controls with data on waist, p* |
| --- | --- | --- | --- | --- | --- | --- | --- |
| Age at examination (years), mean (SD) | 53.1 (9.4) | 51.5 (9.1) | 53.0 (9.4) | 51.6 (9.0) | *0.066* | *0.008* | *0.891* |
| Calendar year at examination, n (%) |  |  |  |  | *<0.001* | *<0.001* | *1.000* |
| 1986-1990 | 11 (8) | 31 (7) | 33 (8) | 94 (7) |  |  |  |
| 1991-1995 | 2 (1) | 170 (41) | 6 (2) | 511 (41) |  |  |  |
| 1996-2000 | 31 (23) | 154 (37) | 93 (23) | 460 (37) |  |  |  |
| 2001-2005 | 54 (39) | 64 (15) | 158 (39) | 196 (16) |  |  |  |
| 2006-2010 | 39 (28) | 0 (0) | 117 (29) | 1 (0) |  |  |  |
| 2010-2011 | 1 (1) | 0 (0) | 2 (1) | 0 |  |  |  |
| Female sex, n (%) | 95 (69) | 284 (68) | 279 (68) | 858 (68) | *0.834* | *0.951* | *0.490* |
| Body mass index (kg/m^2^), mean (SD) | 27.1 (5.1) | 26.0 (4.0) | 26.1 (4.2) | 25.8 (4.0) | *0.020* | *0.231* | *0.028* |
| BMI category, n (%) |  |  |  |  | *0.003* | *0.469* | *0.009* |
| Underweight | 2 (1) | 3 (1) | 5 (1) | 10 (1) |  |  |  |
| Normal weight | 52 (38) | 168 (40) | 175 (43) | 573 (46) |  |  |  |
| Overweight | 47 (34) | 184 (44) | 167 (41) | 494 (40) |  |  |  |
| Obese | 37 (27) | 61 (15) | 62 (15) | 164 (13) |  |  |  |
| Smoking habits, n (%) |  |  |  |  | *0.703* | *0.006* | *0.001* |
| Non-smoker | 48 (35) | 129 (31) | 183 (46) | 629 (51) |  |  |  |
| Ex-smoker | 44 (32) | 131 (32) | 148 (37) | 350 (28) |  |  |  |
| Smoker | 46 (33) | 151 (37) | 71 (18) | 256 (21) |  |  |  |
| Educational level, n (%) |  |  |  |  | *0.012* | *<0.001* | *0.095* |
| 9 years or less | 46 (33) | 173 (42) | 98 (24) | 440 (36) |  |  |  |
| 10-12 years | 58 (42) | 181 (44) | 201 (49) | 556 (45) |  |  |  |
| More than 12 years | 34 (25) | 58 (14) | 109 (27) | 237 (19) |  |  |  |
| Positive ACPA at examination, n (%) | 54 (47) | 125 (35) | 8 (6) | 12 (2) | *0.020* | *0.010* | *<0.001* |
| Time to RA symptom onset (years), mean (SD) | 4.6 (3.6) | 7.0 (4.6) |  |  | *<0.001* |  |  |
| Age at RA symptom onset (years), mean (SD) | 57.6 (9.8) | 58.5 (10.7) |  |  | *0.409* |  |  |
| Calendar year at symptom onset, n (%) |  |  |  |  | *<0.001* |  |  |
| 1986-1990 | 1 (1) | 1 (0) |  |  |  |  |  |
| 1991-1995 | 4 (3) | 32 (8) |  |  |  |  |  |
| 1996-2000 | 7 (5) | 111 (27) |  |  |  |  |  |
| 2001-2005 | 22 (16) | 137 (33) |  |  |  |  |  |
| 2006-2010 | 78 (57) | 116 (28) |  |  |  |  |  |
| 2010-2011 | 26 (19) | 22 (5) |  |  |  |  |  |
| Positive ACPA at/after RA diagnosis, n (%) | 92 (78) | 288 (81) |  |  | *0.504* |  |  |
| Positive RF at/after RA diagnosis, n (%) | 103 (76) | 331 (79) |  |  | *0.469* |  |  |
| Positive ACPA and/or RF at/after RA diagnosis, n (%) | 112 (82) | 362 (87) |  |  | *0.261* |  |  |
|  |  |  |  |  |  |  |  |

Waist circumference was measured in the MONICA cohort, and was introduced as a measurement in the VIP cohort 2004.

SD = standard deviation, BMI = body mass index, ACPA = anti-citrullinated peptide antibodies, RA = rheumatoid arthritis, RF = rheumatoid factor
